# Fevers and Infectious Diseases

**Published:** 1894-06-16

**Authors:** 


### Medical Progress.

### FEVERS AND INFECTIOUS DISEASES.

**On the Production of Fever.**—Hale White,[1] writing on the theory that all pyrexia is essentially neurotic, considers that a rise of temperature may be due either to direct interference with the thermic centres or to the circulation through them of toxic blood or to reflex stimulation of them. As instances of the last case, he gives the pyrexia seen in gallstone colic, in simple fractures, and where pus under tense fasciæ causes effects far out of proportion to its quantity. Specific toxins, or full doses of caffeine or belladonna, causes pyrexia through the blood. As to direct action, experimental injury to the corpus striatum in rabbits causes pyrexia, while injury to the cortex an erratic and irregular form. He considers the thermogenetic centres are situated in the former, and the thermotaxic in the latter. Clinical records bear out this view, and he notices that if one corpus striatum be injured the temperature is usually higher on the opposite side of the body, and that the pyrexia from a striate lesion attains its maximum in twenty-four hours, and is followed by a subnormal fall. In brain hæmorrhage there may be a primary fall from shock. Perversion of the thermotaxic mechanism is shown in laceration of the cortex, meningeal hæmorrhage, and meningitis. In the latter the temperature is erratic, and may reach a high degree, but it bears no relation to the pulse and respiration. Hence, probably, the amount of heat produced by the body is not correctly expressed by the rise of temperature. In epilepsy, chorea, and hysteria there is functional interference with the cortex, and in each disturbance of the temperature may occur. This is marked in the status epilepticus. A rise is found at times immediately after epileptic fits, and when occurring in hysteria the temperatures are erratic, are often not related to the pulse and respiration, are affected very little by antipyretics, and may vary in different parts of the body. Injuries of the cord and pons produce pyrexia by interference with the transmitting fibres, and finally those pyrexiæ produced through the thermolytic centres (vasomotor and sweat) are well recognised.

C. C. Easterbrook[2] defines pyrexia as a morbid elevation of temperature, and fever as a derangement of the quantity of heat in the body. Either can exist without the other. Heat production depends on the nerve control of metabolism. The theory[3] that fever depends essentially on an increase of heat production has been advanced by Hiller, while Kraus, of Vienna, claims to have proved by calorimetry that a rise of temperature is accompanied by diminished radiation, and that the vessels of a fever patient are alternately contracted and dilated. Occasionally fevers, such as scarlet[4] or typhoid, run their course with subnormal temperatures. Teissier[5] relates a case of pneumonia of this kind, and thinks that such instances are due to the retention of the products of cellular nutrition, under the influence of the specific poison. An analagous case is presented by the subnormal temperatures sometimes found in gout. Dr. Teissier employs quinine in these interesting cases, and remarks that it raises the temperature. D'Arsonval[6] and Charrin[7] lay down that (1) microbes act by their products both on the central heat and radiation, increasing the one and diminishing the other. (2) Pyrexia may be caused by substances producing vaso constriction, as well as by those producing vaso dilatation, pyocyanic toxins, and tuberculin. Hence the inadequacy of a vasomotor theory of fever. (3) Calorimetric observations can alone show the pathology of fevers. Over production of heat may exist while the thermometer shows either a great fall or rise of temperature. (4) They have found substances which diminish production of heat and yet raise peripheral temperatures, and others actually increase the temperature while they increase radiation.

The ordinary hectic fever is universally associated with the presence of pus generating organisms, and is characterised by a cold, a hot, and a sweating stage. The regularity of these stages is important in the diagnosis of hectic, but the quantity of the perspiration bears a very variable relation to the severity of the disease or to the height of the temperature. The difficulties of diagnosis with regard to malarial diseases is discussed in a paper by H. S. Stark, of New York[8]. As to pathology, he considers it to be the result of the poisoning of nerve centres by the products of strepti and staphylococci. This nerve element is shown in the flush and sweats, which are such marked features of the disease, and are typical vaso-motor effects also.

An important paper on nerve debility in the production of fever was read at the International Congress by Professor Bouchard,[9] who calls attention to the rise of temperature caused in debilitated persons by sudden emotion, by the first meal of solid food in an illness, the visits of friends, or removal to a hospital; and holds that these are simply instances where, owing to the instability of the nervous system, the heat producing or destroying functions are easily set in motion. From the experiments of Liebermeister and himself he believes that even moderate muscular effort would produce a great rise of temperature were it not controlled by the thermotaxic mechanism, which is both prophylactic and curative. Some rise even in health is produced by any exercise of the muscles, but this is quickly checked by automatic action. Thus external cold produces shivering and muscular tremor, with destruction of cell contents, tending to increase the production of heat, and, on the other hand, it contracts the superficial vessels and stops evaporation from the sweat glands. While reflexes thus defend us from external cold and heat, the internal heat is regulated directly by the nervous centres, as their own temperature is affected by the temperature of the deeper parts of the body. Whenever, then, the nervous system is weakened the ordinary forces which produce or dissipate heat are seen as under a magnifying glass. Thus we may have pyrexia from muscular work, emotion, or even from intellectual effort, when the ordinary restraining forces are in abeyance.

In a paper on hyperpyrexia Dr. Willoughby[10] argues that abnormally high temperatures must be controlled to prevent rapid granular degeneration of the heart and other tissues, either by drugs which decrease oxidation and favour heat elimination, or by the application of cold. The latter is by far the most successful, not only regulating heat, but soothing the injured nervous system.

The number of fevers which are found to be of bacterial origin is constantly increasing, and great interest has been created by the proof of the influence of light in destroying microbic life. Professor Marshall Ward[11] shows that the blue rays of the spectrum are those which possess this bactericidal power, but Khmelevsky found that retardation was produced even by electrical light, and that the effect of solar rays varies with the species of microbes exposed to them. It is disputed whether thermal rays have this power, and the possibility of utilising sunlight as a disinfectant for articles like leather, which are damaged by moist heat, has been disproved by the elaborate experiments of Von Esmarch.[12]

[1] Med. Week., Ap. 20, 1894. [2] Ed. Med. J., May, 1894. [3] Am. J. Med. Sc., March, 1894. [4] Med. Week., March 30, 1894. [5] Journ. des Praticiens, Feb. 7, 1894. [6] Med. Week., Feb. 3, 1894. [7] Med. Week, Mar. 9, 1894. [8] Med. Rec., Ap. 14, 1894. [9] Med. Week., Ap. 6, 1894. [10] Lancet, March 10, 1894. [11] B. M. J., May 5, 1894. [12] Zeit. J. Hyg. xvi., B 2.

